# UK medical students graduating early to work during the COVID-19 pandemic

**DOI:** 10.1017/S0033291720001488

**Published:** 2020-05-07

**Authors:** Shazia P. Sharif

**Affiliations:** Honorary Consultant Paediatric Surgeon, The Royal London Hospital, Barts Health NHS Trust, Whitechapel Road, London, E1 1FR, UK

Dear Editors,

I write in response to ‘Urgent need to develop evidence-based self-help interventions for mental health of healthcare workers in COVID-19 pandemic’. I wholeheartedly agree with the authors on their sentiment to provide urgent psychological support for healthcare professionals. I also write to highlight a potentially vulnerable group: the newest cohort of doctors.

My students are optimistic, intelligent and eager to help by working in the NHS during the COVID-19 pandemic. The majority of them have worked strenuously for 5 or 6 years at the university to get to this position. This has entailed hard work, late nights and personal sacrifices: they are now officially doctors. However, the finish line does not look as expected – missed electives abroad, changed examinations and online graduation ceremonies. They are considering carefully the option of starting work early as an FiY1 doctor or waiting until the traditional August date. As those that have opted to work early, await emails to confirm their placement details, they are experiencing juxtaposed emotions. Pride and anticipation stand next to anxiety and unspoken doubts.

Medical schools, the General Medical Council and Health Education England (HEE) have all rapidly produced different induction packages to get new doctors ready. Starting a new, high-pressure medical job in the midst of a pandemic is new territory that we have little experience of. How are these new doctors feeling? Are there certain types of induction training which will put them on a more secure footing now, and in the long-term? On the 17th of April 2020, a statement from HEE highlighted that ‘…3,000 medical students have chosen to graduate early, freeing them up to start work over the next few weeks and months instead of in August’. In total there are almost 25 000 student doctors, nurses and midwives who have opted to join the workforce early.

The 2012 MERS outbreak in Saudi Arabia had a significant impact on the area. Al-Rabiaah et al. (2020) have recently reported on their findings of anxiety in medical students during this outbreak and the possible major predictors of increased anxiety. They highlighted the need to develop a psychological support programme for medical students during an outbreak of an infectious disease. There is also work by Wong et al. (2004) carried out during the 2003 SARS outbreak in Hong Kong. This shows that nursing students were considerably more stressed than medical students. It is postulated that the perceived prolonged patient contact time nursing care entails may be the basis for this.

We have started the ACED (*ACE*lerated *D*octor) Study to assess the mental health and induction preparedness of medical students as they start their first job. This survey will be repeated in 6 months and again in 1 year with the same cohort of doctors. World Health Organization (WHO) have highlighted the importance of psychological support during the pandemic. UK Hospital trusts are considering what psychological support measures need to be put into place for healthcare professionals after the pandemic is contained. We need to actively consider how doctors, and other healthcare professionals, who first started work during the pandemic will fare psychologically. In particular, the long-term impact and outcomes and how these will be measured.
